# Thyroid Hormone and the Neuroglia: Both Source and Target

**DOI:** 10.4061/2011/215718

**Published:** 2011-08-23

**Authors:** Petra Mohácsik, Anikó Zeöld, Antonio C. Bianco, Balázs Gereben

**Affiliations:** ^1^Laboratory of Endocrine Neurobiology, Institute of Experimental Medicine, Hungarian Academy of Sciences, Budapest, H-1083, Hungary; ^2^Division of Endocrinology, Diabetes, and Metabolism, University of Miami Miller School of Medicine, Miami, FL 33136, USA

## Abstract

Thyroid hormone plays a crucial role in the development and function of the nervous system. In order to bind to its nuclear receptor and regulate gene transcription thyroxine needs to be activated in the brain. This activation occurs via conversion of thyroxine to T3, which is catalyzed by the type 2 iodothyronine deiodinase (D2) in glial cells, in astrocytes, and tanycytes in the mediobasal hypothalamus. We discuss how thyroid hormone affects glial cell function followed by an overview on the fine-tuned regulation of T3 generation by D2 in different glial subtypes. Recent evidence on the direct paracrine impact of glial D2 on neuronal gene expression underlines the importance of glial-neuronal interaction in thyroid hormone regulation as a major regulatory pathway in the brain in health and disease.

## 1. Introduction

Thyroid hormone is a fundamental regulator of biological processes, including cell proliferation, differentiation, and metabolic balance [1]. Thyroid hormone plays a crucial role in brain development, which is illustrated by the dramatic neurologic impairment observed in untreated neonatal hypothyroidism, a condition leading to cretinism [2–4]. The adult brain is also sensitive to thyroid hormone in view of mood disorders, depression, memory, cognitive and motoric impairments frequently observed in hypothyroid patients [5]. 

The major secretory product of the human thyroid gland is thyroxine (T4), a prohormone that does not efficiently bind thyroid hormone receptor (TR). T4 has to be converted to 3,5,3′-triiodothyronine (T3) in order to bind to TR and initiate thyroid hormone-mediated changes in gene expression profiles. Notably, a significant amount of brain T3 is derived from the local activation of prohormone T4 to T3 (80% in the cortex), suggesting that most T3 acting in the brain is generated *in situ* from T4 deiodination [6]. Plasma T3 was also shown to enter the brain [7], and studies on the monocarboxylate transporter 8 (MCT8) knock-out mice indicate that MCT8 plays an important role in this process, but other transporters might also be involved [8, 9]. However, only supraphysiological doses of T3 were sufficient to suppress pro-TRH mRNA in the hypothalamic paraventricular nucleus of hypothyroid rats [10] indicating that T4 uptake into the brain is important for normal function of T3-mediated processes in this tissue. Further studies are required to better understand the transport of different thyroid hormone derivatives across the blood-brain and CSF-brain barrier, the consequences of this mechanism, and the factors affecting this process (see also Section 3.3).

Local T3 generation in the brain is catalyzed by the type 2 deiodinase (D2), a tightly controlled selenoenzyme [11–14]. D2 is the only known protein capable of producing T3 in the human brain [15]. Beyond D2-mediated T3 generation, type 3 deiodinase (D3) is also similarly important for thyroid hormone economy in the brain [16, 17]. D3 inactivates T3 and converts T4 to reverse-T3 that cannot bind to TR. Thus, in contrast to D2, D3 catalyzes the inactivation pathway of thyroid hormone metabolism. 

D2 is expressed in glial cells, including astrocytes in different brain regions and tanycytes, the specialized glial cells in the walls and floor of the third ventricle of the mediobasal hypothalamus [18–20]. In contrast to the glial D2, D3 expression in the brain is restricted to neurons [21]. While historically glial elements of the brain were viewed as a type of connective tissue of the CNS without any real function, this view was overturned by the abundant data on the complex role of glial cells in brain metabolism [22]. According to this more recent hypothesis, glial D2 provides T3 for neighboring neurons that express TR but lack T3 generating capacity [4, 19, 20, 23–25]. 

Thyroid hormone exerts its biological effects predominantly via binding to its nuclear TR, but specific nongenomic effects have also been suggested [26–29]. Two TR isoforms, *α* and *β*, act as ligand-regulated transcription factor and have a central role in transducing the hormonal signal into a cellular response in the brain (reviewed in [30, 31]). Despite accumulating evidence demonstrating that thyroid hormone alters astrocytes function (see Section 2), the presence of TR in astrocytes remains controversial.

The presence of TR in astrocytes has been suggested by *in vitro* studies [32–34], but lower receptor concentrations have been detected compared to oligodendrocytes or neurons [34]. The presence of TR could also be detected in purified glial nuclei from postnatal rat brain [35]. In contrast, *in vivo* data suggested that thyroid hormone would mediate astrocyte function indirectly, based on the lack of immunofluorescence staining of TR receptor isoforms *α*1, *β*1, and *β*2 in GFAP positive astrocytes of the adult rat brain [36]. Interestingly the same group used immunofluorescent to locate TR in cultured astrocytes [37], in line with other *in vitro* data. Astrocytes from distinct developing brain regions are differently responsive to thyroid hormone, with the highest sensitivity in the hemispheres [38]. Thyroid hormone has been shown to be essential for maturation of rat cerebellar astrocytes [39], and TR*α*1 knock-out mice display astrocyte maturation defects suggesting the role of this TR isoform to mediate a direct effect of thyroid hormone action in astrocytes [40]. Presently, most studies agree with the presence of TR*α*1 isoform in astrocytes; discrepancies remain in the case of TR*β* receptor subtypes. Expression of different TR isoforms has been reported in human astrocytomas [41]. Thyroid hormone action occurs within limited time windows, a spatially and timely controlled phenomenon. Cultured cells and most astrocytomas are devoid of the same control conditions, which normally act on cells in the brain, and this could be a background of the different experimental results obtained between *in vitro* and *in vivo *data. In addition, cultured astrocytes are most likely reactive astrocytes, and the heterogeneity of the *in vitro* experimental results regarding TR expression in these cells may reflect the different experimental conditions, such as the brain region and the age of the animals used for cultivation or the different culture conditions.

Active transport of thyroid hormone into brain cells adds to the complexity of thyroid hormone economy in the central nervous system [42, 43]. The MCT8 (SLC16A2) and organic anion transporter 1C1 (OATP1C1) are the best studied thyroid hormone transporters [44, 45]. MCT8 seems to be the predominant neuronal T3 transporter, and its mutations are associated with the Allan-Herndon-Dudley syndrome characterized by congenital hypotonia that progresses to spasticity with severe psychomotor delays [44, 46, 47]. OATP1C1 has high affinity to T4 and is expressed in brain endothelial cells and also in vascular end-feet of astrocytes [48]. Interestingly, tanycytes seem to coexpress MCT8 and OATP1C1 [48]. Other thyroid hormone transporters have also been identified including MCT10, which seems to transport T3 more effectively than MCT8 [49] and the L-type amino acid transporters (LATs) [50]. MCT10 expression was demonstrated in microglia while LAT1 and LAT2 expression was found both in astrocytes and neurons; LAT2 was also present in microglia [51]. Studies on the MCT8 deficient mouse revealed that in the absence of functional MCT8, alternative thyroid hormone transporters play an important complementary role in neuronal T3 transport. In contrast, the lack of alternative pathways, for example, LAT2 in developing human neurons, might be involved in the devastating neurodevelopmental phenotype seen in MCT8-deficient patients with Allan-Herndon-Dudley syndrome [8, 52].

Studies on transgenic mice with targeted inactivation of different members of the deiodinase enzyme family, MCT8, or their combined deletion provided important information on the complex nature of functional interactions between factors regulating thyroid hormone metabolism and transport in the brain and other tissues [8, 9, 53–58]. Despite the relatively mild brain phenotype of D2KO or MCT8KO mice, their combined inactivation led to aggravated manifestation of thyroid hormone deprivation and resulted in similar effects as observed in hypothyroidism [9, 59].These data confirmed the crucial role of D2 in local T3 generation in the brain and suggested that changes in D2 expression could compensate for defects in MCT8 function in the rodent brain.

Numerous aspects of deiodinase-mediated changes of thyroid hormone metabolism have been carefully reviewed and provide a comprehensive view on the molecular and biochemical properties, structure, regulation, and biological functions of these enzymes in the brain and different tissues [24, 60–69]. In the present paper we will focus on the role and regulation of thyroid hormone in neuroglia, representing an exciting aspect of emerging significance for thyroid hormone economy. We provide a concise overview on the most important effects of thyroid hormone on glial cells, followed by the discussion of novel data on D2-mediated glial T3 generation and its role under specific physiological and pathophysiological conditions.

## 2. Thyroid Hormone-Mediated Changes in Neuroglial Cells

Brain development provides the best studied model of thyroid hormone action in the brain. It has been known for decades that hypothyroidism can result in numerous brain defects, including decreased dendritic arborization of Purkinje cells, diminished axonal outgrow and myelinization, and insufficient cortical layer organization [70]. Although thyroid hormone also impacts the adult brain, the underlying cellular and molecular events are less understood [71, 72]. Various aspects of thyroid hormone-mediated brain function were extensively reviewed (see Section 1).

Available data on thyroid-hormone-regulated gene networks are yet limited, but accumulating evidence indicates that various sets of genes are regulated along this pathway. In a recent study, thyroid hormone action on adult rat striatum was monitored using gene expression profiling [73]. The numerous up or down regulated sets of genes involved various pathways affecting for example, circadian regulation, oxidative stress response, phenylethylamine degradation, MAPK pathway, phosphate metabolism, signal transduction, and cell structure. These findings revealed novel aspects of brain related thyroid hormone action that need to be studied in details. Numerous examples of thyroid hormone-dependent gene expression in the brain are related to neurons, which could be a result of direct neuronal effect or an indirect glia-mediated signal [74]. Neuroglial cells are heavily involved in the regulation of neuronal metabolism and activity, glucose supply, cerebral blood flow, and neurotransmitter levels in mature brain [22]. The detailed description of mechanisms how glial cells are involved in this process is an ongoing effort and requires further studies. We will briefly summarize below how thyroid hormone targets glial cells and mediates their function that has also consequence on glia-mediated neuronal activities.

### 2.1. Differentiation, Maturation

Thyroid hormone affects the differentiation and maturation of different glial subtypes including astrocytes, oligodendrocytes, and microglia [75–77]. Although many aspects of glial linages are yet controversial, evidence has been obtained *in vitro* that oligodendrocytes and astrocytes are derived from a common precursor, the glial restricted precursor cells (GRP) [78]. GRPs are tripotential cells, owning the ability to divide into myelin producing oligodendrocyte or two types of astrocytes, depending on the factors contained in growth medium. *In vitro* studies demonstrated that mature oligodendrocytes were developed from precursor cells in the presence of thyroid hormone and platelet-derived growth factor (PDGF) [79]. The concept that these two glial cell types originate from a common lineage was also supported by findings that show reciprocal changes in oligodendrocyte/astrocyte cell density in the rat white matter upon changes in serum T4 level [80]. Furthermore, the number of matured oligodendrocytes and astrocytes was reduced in the brain of hypothyroid animals within white matter tracts [81, 82]. Morphological differentiation of astrocytes from progenitors to mature cells has been explained by thyroid hormone-mediated actions affecting cytoskeletal proteins (F-actin, GFAP) [40, 83]. In hypothyroid neonatal rats, there was reduced GFAP content in hippocampus and basal forebrain [84]. In cell cultures, T3 upregulates GFAP production and reorganizes GFAP filaments and transforms the flat polygonal astrocytes into process-bearing cells [85, 86]. Not only T3, but T3-mediated growth factor secretion can enhance GFAP expression, thanks to several growth factor binding domains in its promoter region [87, 88]. 

Microglial cell development is also affected by thyroid hormone. In the cortical forebrain of hypothyroid neonatal rat there is diminished amount of cell bodies and less abundant microglial process density. T3 favored survival of microglias *in vitro* and have triggered their process extension [77, 89]. 

The mechanisms by which thyroid hormone promotes differentiation were not yet fully revealed, but there are several candidates for this process, for example, cell-cycle modulators like E2F-1, cyclin D1, and p27 [90]. E2F1 is a key transcription factor, that controls G1 to S phase transition and has an impact on cyclin D1 [91]. E2F-1 and cyclin D1 expression is down regulated via TR-mediated transcriptional repression [92, 93]. Another candidate of this pathway, p27 cyclin-dependent kinase inhibitor was upregulated in response to T3 [94, 95]. Decreased amount of E2F-1 and cyclin D1 protein and increased levels of the p27 cell-cycle inhibitor may shift cell fate towards differentiation.

### 2.2. Myelination

Myelination represents the best characterized T3-dependent glial action in the brain [96–98]. Thyroid hormone regulates oligodendrocyte differentiation and myelin production via TR-mediated transcriptional effects [34, 99]. Thyroid hormone depletion resulted in delayed expression of oligodendrocyte-specific markers [100] and decreased the number of oligodendrocyte cell bodies in the main white matter tracts [81]. Hypothyroidism delayed the expression of genes encoding structural proteins of myelin, for example, myelin basic protein (MBP), proteolipid protein (PLP), and myelin-associated glycoprotein (MAG) [101] and resulted in reduced numbers of myelinated axons and lower myelin content [102]. Sensitivity period of these genes for thyroid hormone extends from the end of the first postnatal week up to the end of the first month in rat [76, 103].

### 2.3. Extracellular Matrix Formation and Cytoskeleton Organization

Thyroid hormone action on astrocytes during brain development is illustrated by enhanced secretion of extracellular matrix proteins and growth factors. Astrocytes were previously shown to produce laminin and fibronectin [104, 105]. Subsequent studies demonstrated T3-induced expression of laminin and fibronectin in cultured cerebellar astrocytes and revealed that both laminin and fibronectin were organized in fibrillar pattern on the cell surface, while hypothyroid conditions changed this distribution for a disorganized extracellular matrix of punctuate pattern [106]. As an underlying mechanism it was suggested that astrocytes modulate extracellular matrix composition via T3-mediated growth factor secretion [104, 107]. 

Basic fibroblast growth factor (bFGF) and epidermal growth factor (EGF) are secreted by cerebellar astrocytes in response to T3 and seem to promote extracellular matrix protein secretion and organization in an autocrine manner [106]. EGF was suggested to exert its effect on extracellular matrix protein secretion through MAPK/phosphatidylinositol 3-kinase pathway [108]. Astrocytes also secrete nerve growth factor (NGF) in a T3-dependent manner, which allows potent control of neurite growth and survival [109–111].

Beyond T3, the effect of T4 on astrocytes was also demonstrated suggesting that thyroid hormone could also impact astrocytes via a nongenomic pathway. T4 exerts its effect on the microfilament network of astrocytes by dynamically organizing F-actin filaments, facilitating integrin clustering, and focal contact formation [112, 113]. Polymerized actin filament network was observed in cultured astrocytes after treatment with T4 and reverse T3 while T3 did not affect the polimerization rate [114, 115]. 

Adhesive interactions among the extracellular matrix protein laminin, integrins, and the microfilament network play a fundamental role in the regulation of neural cell migration during brain development.* In vitro* studies on neurite development demonstrated that neurons, cocultured with astrocytes under thyroid hormone-depleted conditions, showed reduced total neurite length and decreased neurite numbers [108]. As a consequence, these data suggest that thyroid hormone-mediated actions on astrocytes are important events in neuron migration and axon formation.

## 3. Thyroid Hormone Activation in Neuroglia

### 3.1. Regulation of Type 2 Deiodinase in Glial Cells

While thyroid hormone impacts glial function in various manner (see above), neuroglia is not only target but also the predominant source of T3 in the brain. As mentioned above, astrocytes and tanycytes express type 2 deiodinase (D2), the enzyme catalyzing thyroid hormone activation. Below, we will discuss factors and conditions affecting D2 regulation in glial cells, since they can contribute to the better understanding of thyroid hormone signaling in the brain.

#### 3.1.1. Thyroid Hormone

D2 is negatively regulated by thyroid hormone, through a mechanism that involves product (T3-) mediated transcriptional downregulation of the *Dio*2 gene and substrate (T4-) induced posttranslational decrease of D2 protein levels (reviewed in [66], see Section 3.2). The negative regulation of D2 activity suggests a homeostatic regulation of T3 generation [116–118]. However, region-specific differences within the brain regarding the response to hyper- or hypothyroidism are reflected by changes in D2 regulation. D2 is reciprocally regulated by thyroid hormone in various brain regions but shows only modest response in the hypothalamus [19, 119–121]. The fact that D2 activity in the hypothalamus is concentrated in tanycytes [19] suggests marked differences between astrocytes and tanycytes regarding thyroid hormone response and balance. While in astrocytes T3 production seems to serve homeostatic purposes, the relative insensitivity of D2 to T3 in tanycytes would indicate that other signals act more importantly on D2 expression, thus controlling local T3 production [25]. The mechanisms responsible for this difference between astrocytes and tanycytes remain to be determined. However, a link has been suggested between the developmental state of astrocytes and their responsiveness to thyroid hormone [38]. Although tanycytes are still considered as terminally differentiated cells, data have been accumulating that at least a subpopulation of this inhomogeneous cell layer might behave as progenitor cells. This is supported by observations that the tanycyte layer in the wall of the third ventricle regenerates in two weeks following alloxan-induced destruction [122], and tanycytes could be considered a neurogenic niche in response to IGF-I [123]. This is presently unclear whether differences in differentiation stages or a more specific factor is responsible for the different responsiveness to thyroid hormone of the two cell types.

#### 3.1.2. Infection, Nonthyroidal Illness

**Figure 1:**
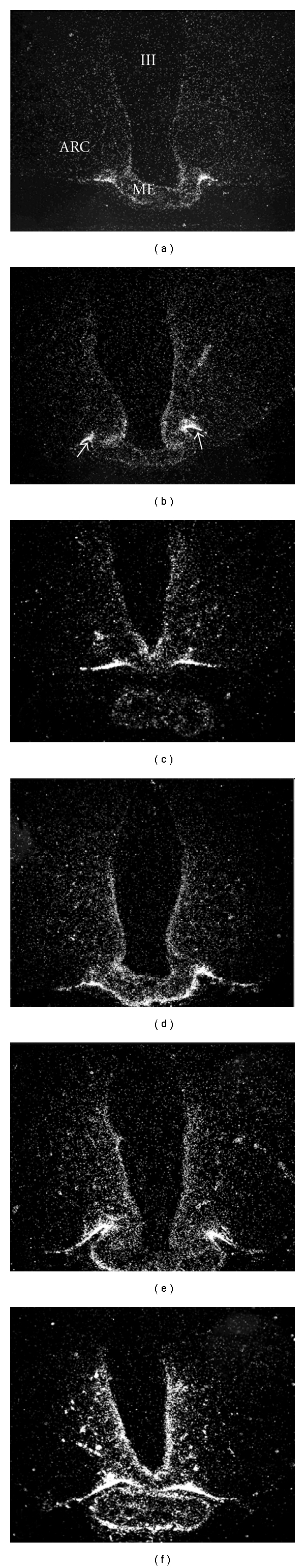
Infection upregulates D2 expression in tanycytes of the rat mediobasal hypothalamus. Dark-field micrographs from three different rostrocaudal levels of the median eminence (ME) showing the effect of *i.p.* LPS treatment on D2 mRNA expression. (a)–(c), Controls; (d)-(f), LPS-treatment. Note: D2 *in situ* hybridization signal is increased in the tanycytes lining the wall of the third ventricle (III), and in the tanycyte processes in the tuberoinfundibular sulci (arrows), in the external zone of the ME. ARC, arcuate nucleus. Reprinted with permission from Fekete et al. [132], The Endocrine Society.

It has been suggested that D2-generated T3 in tanycytes of the mediobasal hypothalamus could play a role in the pathogenesis of nonthyroidal illness during infection [63, 66, 67, 124]. Nonthyroidal illness syndrome (euthyroid sick syndrome or low T3 syndrome) is accompanied by low T3 and sometimes low T4 serum levels and associated with nonelevated or inappropriately elevated TSH levels during infection, sepsis, starvation, malignancy, life-threatening trauma, and other critical illness [125–128]. Although the syndrome has been known for decades, it is still a matter of debate whether the changes of thyroid hormone profile provide physiologic compensation for illness or it represents pathological conditions [129–131]. Systemic administration of bacterial lipopolysaccharide (LPS) increased D2 mRNA expression in tanycytes and D2 activity in the rat mediobasal hypothalamus (Figure 1) accompanied by falling serum thyroid hormone and TSH levels [132]. This phenomenon was also observed in mice, immediately followed by decreased expression of thyroid receptor *β*2, TSH*β* in the pituitary and decreased type 1 deiodinase mRNA in the pituitary and liver [133].

**Figure 2:**
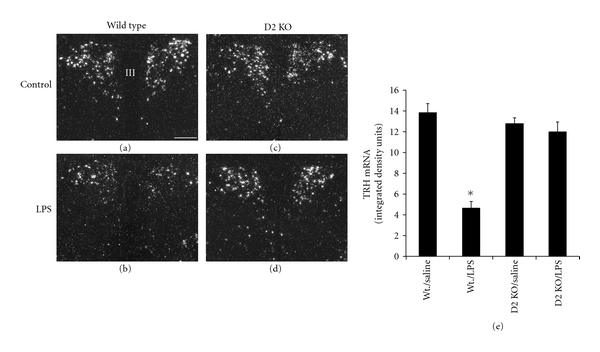
LPS-induced infection downregulates TRH mRNA expression in the hypothalamic paraventricular nucleus of wildtype but in D2 knock-out mice. (a,b), wild-type; (c,d), D2 KO mice; (a,c), control; (b,d), *i.p.* LPS-treated animals; (e), quantification of the TRH mRNA signal by densitometry. Printed from Freitas et al. American Society for Clinical Investigation [23].

LPS induced suppression of TRH expression in the hypothalamic paraventricular nucleus of wild type but the effect was abolished in the D2 knock-out mice (Figure 2) (see Section 3.3) [23]. Although this model is not suitable to dissect the role of specific glial subtypes in this mechanism, it clearly demonstrated the fundamental role of D2 activity in TRH suppression during infection and supported the hypothesis of a close interaction between neurons and glial cells and their role in regulating brain functions via T3 availability. Importantly, while the continuous increase in D2 activity of cortical astrocytes seemed to be the consequence of falling T4 levels, D2 activation in tanycytes followed kinetics that was independent of thyroid hormone levels [132, 134]. It was also demonstrated that LPS-induced D2 expression on the mediobasal hypothalamus was not dependent on circulating corticosterone, either [135]. Unexpectedly, cultured astrocytes of the rat cerebral hemispheres increased their D2 activity in response to LPS, and glucocorticoids enhanced this effect [136]. It is presently not clear why this effect is not reflected *in vivo* by the kinetics of cortical D2 induction. Importantly NF-*κ*B, a potent effector of LPS-induced signaling, transcriptionally activated the D2 encoding *Dio*2 gene, and a functional NF-*κ*B binding site was identified and characterized in the human *Dio*2 5′ flanking region [132, 137]. NF-*κ*B was also involved in the LPS-induced increase in D2 activity in cultured astrocytes [136].

Further studies on the kinetics of LPS-induced activation of the NF-*κ*B pathway in the rat mediobasal hypothalamus indicated that NF-*κ*B activation contributes to sustaining the LPS-induced D2 response in a subset of *α* tanycytes [138]. However, this is not the initiating mechanism of LPS-induced D2 response in tanycytes. The same study suggested that TSH of the part tuberalis could also play a role in this process [138]. The factor(s) that initiate tanycytal D2 induction in the starting phase of LPS-evoked infection are presently not known. However, taking into account the highly active nature of D2-catalyzed T3 generation even a subpopulation of tanycytes could provide a significant amount of T3 for the modulation of TRH expression. To asses this appropriately, it would be important to understand in details the pathways that allow tanycyte-generated T3 to reach hypophysiotropic TRH neurons in the paraventricular nucleus.

#### 3.1.3. Iodine

Iodine availability is critically important to maintain proper thyroid hormone levels. During moderate iodine deficiency most thyroid hormone target tissues are only mildly affected, due to rapid physiological adaptations of the hypothalamo-hypophyseal-thyroid axis, which maintains plasma T3 at the normal range [139, 140]. Via glial D2 and neuronal D3, the brain is capable of adapting to iodine deficiency in a complex manner. A moderately severe iodine deficiency resulted in increased D2 mRNA and activity in different brain regions [140]. D2 sensitivity to iodine deficiency was region specific, the hippocampus and cerebral cortex represented the most responsive regions. D2 induction in this regions indicated that astrocytes increase their T3-generating activity under iodine deficiency. Tanycytes of the mediobasal hypothalamus also increased their D2 expression and activity although their response was lower compared to astrocytes in the cortex and hippocampus. Since increase of D2 activity was higher than that of mRNA expression, it could be speculated that not only pretranslational events are involved here in D2 regulation but also prolonged D2 half-life due to decreased D2 ubiquitination (see Section 3.2) could contribute to this effect. Increased glial D2 in iodine deficiency was paralleled with reduced neuronal D3 [140]. Thus mitigating the effects of iodine deficiency by both increased T3 generation and reduced T3 degradation reflected the particular importance of adaptation of the brain to iodine deficiency. Various aspects of iodine deficiency modulated alterations of thyroid hormone deiodination were extensively reviewed elsewhere [141].

#### 3.1.4. Fasting

D2 expression in tanycytes is modulated by food restriction. Fasting resulted in twofold increase in D2 mRNA expression and activity in rat mediobasal hypothalamus, and it was straightforward to suggest that this could suppress TRH expression in the hypothalamic paraventricular nucleus and downregulate this way the hypothalamo-hypophyseal-thyroid axis. [121]. However, fasting-mediated decrease of TRH expression in the paraventricular nucleus of the TR*β*2-null transgenic mice remained unaffected although TR*β*2 represents the key TR isoform involved in T3-mediated negative regulation of TRH expression in transgenic mice [142]. This finding demonstrated that tanycyte-generated T3 during fasting should not have major direct effects on TRH expression in the paraventricular nucleus. As an alternative pathway, the hypothalamic ventromedial nucleus was also suggested as a target translating changing hypothalamic T3 levels into the modulation of food intake [143]. The role of glial D2-mediated hypothalamic T3 in fasting is not yet resolved, and related data are reviewed elsewhere [25, 61, 63, 66].

#### 3.1.5. Light

D2 expression in the mediobasal hypothalamus is controlled by light, and this has consequences on reproductive function. Light exposure-induced D2 expression in the mediobasal hypothalamus of the Japanese quail (*Coturnix japonica*) represents a crucial event in the signal transduction pathway ensuring photoperiodic response of gonads. Intracerebroventricular administration of T3 mimicked the photoperiodic response, whereas the D2 inhibitor iopanoic acid prevented gonadal growth [144]. Interestingly, beyond median eminence and infundibular nucleus D2 induction was also observed in the dorsal and lateral hypothalamus. Based on this finding it cannot be excluded that not only tanycytes but other cell types, for example, hypothalamic astrocytes could be also involved in this mechanism, but this aspect was not studied in details. A mechanism for light-induced D2 expression in the mediobasal hypothalamus was also revealed in quail showing a preceding peak of TSH*β*-subunit expression in the pars tuberalis via a cAMP-dependent mechanism. It was demonstrated that intracerebroventricular administration of TSH to short-day quail stimulated gonadal growth and D2 expression and proved that TSH in the pars tuberalis therefore seems to trigger long-day photoinduced seasonal breeding [145]. 

A homology between avian and mammalian photoperiodic regulation of reproduction has been observed since D2 expression was also increased in Djungarian (Siberian) hamsters (*Phodopus sungorus*) under long days; the signal was weaker under short days while melatonin injection decreased D2 expression under long days [146]. These results indicate that D2 expression in tanycytes may be involved in the regulation of seasonal reproduction both in mammals and birds. Regulation of seasonal reproduction by photoperiodic regulation of hypothalamic thyroid hormone levels also involves reciprocal changes of D2 and D3 expression that is reviewed elsewhere along with data on other models of seasonal reproduction [66, 147, 148].

#### 3.1.6. Trauma

After traumatic brain injury D2 mRNA was upregulated in reactive astrocytes in rat. In the cerebral cortex near the contusion D2 mRNA was upregulated on the first day after injury; in the following days the signal was shown to have expanded to the hippocampus, where the astrocytic localization of upregulated D2 mRNA was obvious and bordered the neuronal granule cell layer [149]. Furthermore, different stressors including relatively mild ones (e.g., handling) increased D2 activity in a stressor- and brain-region dependent manner [150]. The frontal cortex showed the highest D2 response, and motor stress was the most dominant stressor in this region while no effect was seen in the cerebellum. A stressor-dependent decrease of T4 tissue concentration was also observed but stressor-dependent deviations were also found since, for example, gently handling resulted in elevated T4 in the frontal cortex. A strict correlation between D2 activity and tissue T4 levels could not be found suggesting the role of specific factors and not simply the falling T4 level in stress-related D2 increase [150].

#### 3.1.7. Development

Deiodinases are tightly regulated during various developmental processes (reviewed in [4, 151]). It was shown that the human fetal brain is already sensitive to thyroid hormones before the onset of the fetal thyroid [152, 153]. The presence of high-affinity T3 binding sites with a specificity that resembles that of the nuclear T3 receptors was also demonstrated in the human fetal brain and its concentration increased by tenfold from ten to sixteen weeks [154]. D2 expression and activity was detected in the human fetal cortex already from seven to eight weeks of gestation [155]. It has been also demonstrated that during the second trimester T3 increases in the cortex due to D2 activity, while it remains very low in cerebellum because of D3-mediated thyroid hormone inactivation [156]. Although deiodinase activities were also studied in the developing rat brain, [157], data are limited on ontogenic aspects of D2 expression in different glial subtypes. Increasing D2 expression was detected in the developing chicken brain in perivascular localizations probably localized to glial cells [158]. It was also demonstrated that D2 was expressed in chicken tanycytes before the onset of thyroid hormone-dependent negative feedback. Furthermore, D2 and Nkx2.1 were coexpressed at E13 and P2 in tanycytes but not in the perivascular glia indicating a glial-subtype-specific regulation of D2 expression [159].

#### 3.1.8. Other Factors

It has been demonstrated that D2 expression in glial cells is under the control of multiple factors. These include the increase of D2 activity upon cAMP induction [160, 161] that is in line with the finding of an evolutionary conserved CRE site in the *Dio*2 promoter [61, 162–164]. Selenium dependence [165], phorbol esters and glucocorticoids [166], acidic fibroblast growth factor [167] also impact D2 activity.

### 3.2. Posttranslational Regulation of Glial D2 Activity

It has been demonstrated that D2 activity in the brain undergoes rapid and substrate-induce changes [116, 117]. The underlying mechanism was later identified demonstrating that D2 undergoes substrate-induced ubiquitination followed by its degradation in the proteasome [168–170]. This was a unique example of substrate-induced selective proteolysis that involves ubiquitination of an endoplasmic reticulum resident enzyme and represented the first demonstration that such a regulatory pathway controls activation of a hormone [170]. The pathway works also in primary cultures of astrocytes, since MG132 a proteasome uptake inhibitor could block substrate-induced D2 inactivation [171]. Interestingly, D2 inactivation via ubiquitination does not necessarily involve proteasomal proteolysis. It has been revealed that D2 forms homodimers that undergo ubiquitination-mediated transient and reversible conformation changes. Since dimerization of D2 monomers is crucial to maintain the proper conformation of the active center of the enzyme, ubiquitination-mediated changes result in the rapid loss of D2 activity [172]. 

Since D2 ubiquitination represents a rapid way for the regulation of T3 generation its mechanism was studied in detail in the past several years. UBC6 and 7 were identified as the ubiquitin conjugases (E2) involved in the ubiquitination of D2 [173, 174], while USP-33 and USP-20 (VDU1 and 2) deubiquitinate D2 and prolong its half-life [175]. A novel type of ubiquitination motif containing a 18-aa-loop structure of the D2 protein was identified and characterized [176, 177]. WSB1 (Swip1) was recognized as a sonic hedgehog-induced SOCS-box containing protein of unknown function [178, 179]. Importantly, it could be shown that WSB1 serves as the ubiquitin ligase (E3) that links D2 to the Elongin BC-Cul5-Rbx1 ubiquitinating catalytic core complex [176]. Later, Teb4 has been also identified as a D2 E3 ligase [180]. 

**Figure 3:**
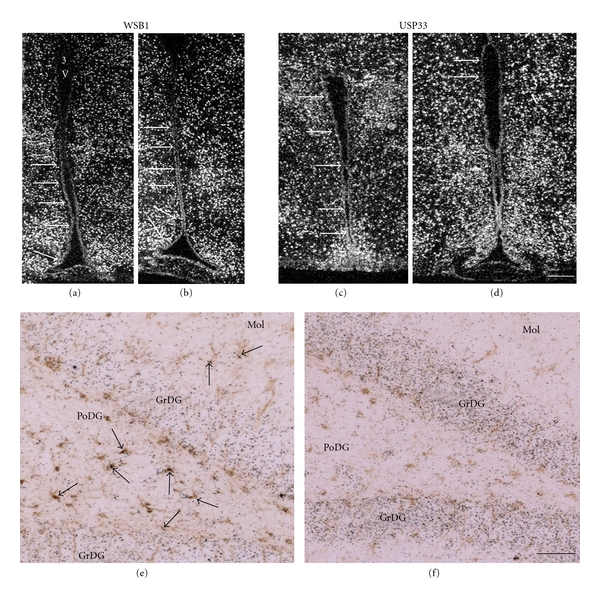
Expression of crucial elements of the D2 ubiquitination machinery in glial cells in the rat brain. (a,b) mRNA of WSB1, the D2 ubiquitin ligase is expressed in tanycytes lining the wall of the third ventricle (3V). WSB1 expression *(arrows)* extended from the anterior lower part (not shown) to the lower two thirds of the wall of the third ventricle in more caudal regions. Neuronal cells also express WSB1. (c,d) Hybridization signal of the USP33 D2 deubiquitinase was also detected over tanycytes and ependymal cells lining all regions of the wall of the third ventricle *(arrows).* Neuronal cells also express USP33. (e) WSB1 *in situ* hybridization signal (arrows) is observed over the majority of GFAP-expressing astrocytes (brown immunoreactivity) demonstrated here in the hippocampal dentate gyrus. (f) The mRNA of USP33 was absent from GFAP-expressing astrocytes (brown) in the hippocampus, but it was expressed in granular neurons. The sense probes for WSB1 or USP33 did not produce any signal (not shown). Mol, molecular layers of the dentate gyrus; GrDG, granular layer of the dentate gyrus; PoDG, polymorph layer of the dentate gyrus. Reprinted with permission from Fekete et al. [181] The Endocrine Society.

Data are accumulating on how crucial elements of the D2 ubiquitination machinery are expressed in D2-expressing glial subtypes. The available data revealed cell-type-specific differences in the expression of crucial elements of the D2 ubiquitinating/deubiquitinating machinery in the rodent brain. WSB1, the D2 E3 ligase, is expressed both in GFAP-expressing astrocytes in different brain regions and in tanycytes in the mediobasal hypothalamus (Figure 3) [181]. This suggested that the WSB1-D2 interaction, a process required for D2 ubiquitination, could be functional in these cells. In contrast to WSB1, the TEB4 E3 ligase could not be detected in GFAP-expressing astrocytes, only in the cerebellum, but it was expressed in tanycytes [180]. Furthermore, the USP33 (VDUI) D2 deubiquitinase is co-expressed with D2 only in tanycytes but not in astrocytes (Figure 3) [181]. WSB1 and USP33 expression in the brain was not affected by thyroid hormone status indicating that these genes are not involved in the homeostatic response to hypo- or hyperthyroidism [181].

The available data suggested that kinetics of D2 ubiquitination and consequent selective proteolysis in the proteasome could be different among different subtypes of glial cells. Among D2-expressing cell types of the brain, tanycytes express the most comprehensive set of genes involved in ubiquitination-mediated D2 regulation, that ensures both WSB1- and TEB4-mediated ubiquitination ligation to D2- and also USP33 deubiquitinase-mediated D2 reactivation. In astrocytes D2 deubiquitinaton is either not possible, or it works via USP20 or other unidentified D2 deubiquitinases.

### 3.3. Neuroglial Thyroid Hormone Metabolism Affects Neuronal Gene Expression

Astrocytes and tanycytes of the neuroglial compartment are the predominant source of T3 present in the brain while TR in neurons represents a major target of thyroid hormone. As discussed above, neurons cannot generate T3 but express type 3 deiodinase (D3), the T3 degrading enzyme. While numerous observations suggested that glial thyroid hormone metabolism could affect neuronal function (see Section 3.1), until recently no direct evidence could be obtained to prove the existence of deiodinase-mediated transcriptional T3 footprints in neurons. Recently a two-dimensional coculture was used based on the D2-expressing H4 glioma cells and the D3-expressing SK-N-AS neuronal cell line. It has been shown that T4 could activate the endogenously expressed T3-sensitive ENPP2 gene of the neuronal compartment only if the glial compartment was present. This model led to the demonstration that D2-mediated glial T3 generation from physiological amount of T4 can directly affect thyroid hormone-dependent gene expression in a paracrine fashion [23]. A different approach using expression profiling-based assessment of thyroid-hormone-regulated gene expression in the cerebral cortex of the MCT8, D2, and MCT8/D2 knock-out mice suggested that negative regulation required D2-generated T3, while peripheral T3 entering the brain should be sufficient to maintain normal expression of positively regulated genes [59].

Specific signals as hedgehog proteins [176, 182], bacterial lipopolysaccharide (LPS) [132, 133, 137, 183], and hypoxia [184] have been established as regulators of deiodinase activities. It was also studied how these specific signals impact neuroglial thyroid hormone metabolism in the coculture system. The sonic hedgehog morphogene decreases glial thyroid hormone activation via WSB1-mediated posttranslational downregulation of D2 (see Section 3.2) and increases neuronal D3 expression [23]. This demonstrates the existence of a mechanism ensuring a fine-tuned balance between sonic hedgehog-mediated proliferation and T3-evoked differentiation. This is interesting since astrocytes are targets of sonic hedgehog signaling [185]. It has been also demonstrated that in the brain T3 upregulates crucial elements of the sonic hedgehog signaling pathway that could represent a compensatory feedback loop for sonic hedgehog-mediated T3 regulation [186]. 

The effect of LPS on D2 expression and its relation to nonthyroidal illness were discussed in Section 3.1. In contrast to sonic hedgehog, LPS-induced glial D2 activity and decreased neuronal D3 in the H4-SK-N-AS system and as a consequence resulted in a decreased T3-mediated gene expression in the neuronal compartment [23]. These data were complemented with *in vivo* observation on the LPS evoked model of nonthyroidal illness. LPS could not induce TRH suppression on the paraventricular nucleus of the D2 knock-out mice only in wild types (Figure 2) (see also Section 3.1). This indicated that glial (highly probably tanycytal) D2-generated T3 in the hypothalamus could play an important role in T3-mediated suppression of the hypophysiotropic TRH neurons and consequently in the decreased activity of the hypothalamo-hypophyseal-thyroid axis during the infection-evoked subtype of nonthyroidal illness [23].

**Figure 4:**
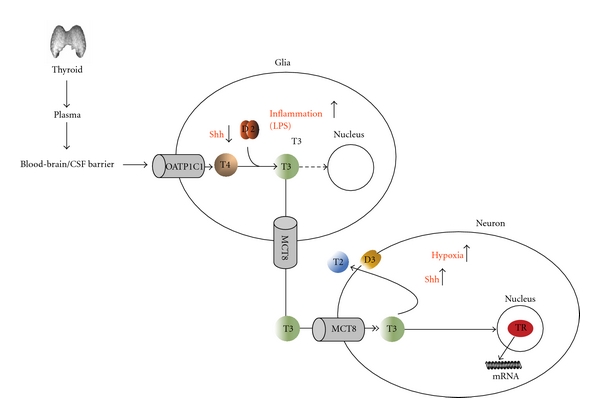
Proposed model of neuroglia-neuron interaction of thyroid hormone signaling in the brain. D2 activates the prohormone T4 in glial cells (astrocytes and tanycytes); the generated T3 exits the glial compartment and enters adjacent neurons, where it establishes a transcriptional footprint via liganding TR. Only the two best-characterized thyroid hormone transporters, OATP1C1 and MCT8, are indicated, but data are also accumulating on the role of LAT1 and LAT2 in the thyroid hormone transport both in neurons and astrocytes (discussed in Section 1). In the glial compartment LPS activates D2 transcription while sonic hedgehog (Shh) promotes D2 inactivation via WSB1—mediated ubiquitination; both hypoxia and Shh activate D3 gene transcription in neurons. Figure modified from Freitas et al. American Society for Clinical Investigation [23].

In contrast, hypoxia affected predominantly neuronal D3 activity in the H4- SK-N-AS system. This effect could be also demonstrated in a rat *in vivo* hypoxia/ischemia model showing D3 induction in cortical neurons and in the hippocampal pyramidal and granular cell layers [23]. This suggested that lowered local T3 levels improve neuronal survival under hypoxic challenge. The glial aspect of this phenomenon requires further studies since an independent study on primary cultures of astrocytes demonstrated hypoxia-induced increase of D2 activity [171]. These data established deiodinase enzymes as glial and neuronal control points for the regulation of thyroid hormone action in the brain during health and disease (Figure 4) [23].

## Abbreviations

D2: type 2 deiodinase
D3: type 3 deiodinase
GFAP: glial fibrillary acidic protein
T4: thyroxine
T3: 3,5,3′-triiodothyronine
TR: thyroid hormone receptor
LPS: bacterial lipopolysaccharide
TSH: thyrotropin
TRH: thyrotropin-releasing hormone.
